# The Efficacy of Yanghe Decoction on Diabetic Foot: A Systematic Review and Meta‐Analysis

**DOI:** 10.1155/ije/9924142

**Published:** 2026-04-30

**Authors:** Xia Wang, Yunjiao Yang, Junhua Pan, Qiu Chen

**Affiliations:** ^1^ Department of Endocrinology, Hospital of Chengdu University of Traditional Chinese Medicine, Chengdu, Sichuan, China, scu.edu.cn; ^2^ School of Clinical Medicine, Chengdu University of Traditional Chinese Medicine, Chengdu, Sichuan, China, scu.edu.cn; ^3^ Dazhou Hospital of Integrated Traditional and Western Medicine, Dazhou, Sichuan, China, scu.edu.cn

**Keywords:** ankle-brachial index, diabetic foot, diabetic foot ulcer, meta-analysis, Yanghe decoction

## Abstract

**Purpose:**

To evaluate the potential clinical effect of Yanghe decoction on diabetic foot.

**Methods:**

We comprehensively searched Web of Science, PubMed, The Cochrane Library, Embase, CNKI, Wanfang, and VIP databases from their inception to December 6, 2025, to identify randomized controlled trials (RCTs) investigating Yanghe decoction for DF. Meta‐analysis was performed using RevMan 5.4 and Stata 15.0 software.

**Results:**

A total of 15 studies with 1224 patients were included, 614 in the treatment group and 610 in the control group. The results suggest that the total effective rate of the treatment group may be higher than that of the control group (RR = 1.23, 95% CI: 1.17 to 1.29, *p* < 0.00001). The wound healing time of the treatment group may be shorter than that of the control group (MD = −9.36, 95% CI: −14.21 to −4.50, *p* = 0.0002), and the treatment group may have potential advantages over the control group in the improvement of wound area after treatment (SMD = −2.57, 95%CI: −3.94 to −1.20, *p* = 0.0002). There was no statistically significant difference in the ankle‐brachial index (ABI) between the treatment group and the control group (MD = 0.05, 95% CI: −0.09 to 0.18, *p* = 0.51). The funnel plot of the total effective rate between the treatment group and the control group suggested that there was a publication bias in the effective rate. Further analysis of the results by the nonparametric trim and filling method suggested that the results of the meta‐analysis were relatively stable, and the possible publication bias did not substantially affect the results.

**Conclusions:**

Low certainty evidence suggests that Yanghe decoction may potentially improve the clinical symptoms of diabetic foot, may shorten the wound healing time, and may reduce the wound area. However, the independent clinical efficacy of Yanghe decoction for diabetic foot cannot be determined due to the high heterogeneity of cointerventions in the included studies. This study is further limited by the insufficient quantity and quality of the included studies, and the above tentative implications need to be verified by more large‐sample and multicenter RCT studies that standardize interventions and control for cointervention variables.

## 1. Introduction

Type 2 diabetes mellitus (T2DM) is linked with numerous microvascular and neuropathic deleterious outcomes throughout the body, encompassing conditions such as diabetic retinopathy and diabetic foot (DF) complications [1–3]. DF is a common chronic complication of diabetes, which refers to infections and ulcers that occur in the local skin of diabetic patients, so it is also called diabetic foot ulcer (DFU). Domestic literature underscores that approximately 15%–20% of diabetic patients are afflicted by DFU, with a striking 31.6% reulceration incidence observed within a year of ulcer recovery [4, 5]. Global statistics reveal an annual tally ranging from 9.1 million to 26.1 million diabetic individuals grappling with DF. Projections indicate an expected diabetic populace surge to 1.31 billion by 2035 [6]. Regrettably, the toll of this malady translates to a diabetic amputation every 20 s, with annual mortality rates reaching an alarming 11% among DF patients and soaring to 22% among amputees [7, 8]. DF predominantly infiltrates deep tissues, oftentimes intertwined with distal vasculature and lower limb neuropathy, culminating in severe cases of foot gangrene [9]. Presently, DF treatment primarily enlists conventional tactics such as surgical debridement, prompt revascularization, anti‐infective interventions, and localized decompression. Nonetheless, treatment efficacy occasionally falls short of expectations, beset by numerous adverse reactions and recurrences [10, 11]. In recent years, traditional Chinese medicine (TCM) has been explored for its potential in DF management, particularly in alleviating its clinical manifestations [12]. TCM identifies Yin and Yang depletion alongside blood stasis as pivotal pathogenic mechanisms underlying DF, categorizing it within the realms of necrosis and vascular paralysis. Clinically, patients frequently manifest intense pain, necrosis, and extremity ulceration. The lingering hyperglycemia, hyperlipidemia, and local microcirculatory insufficiency often lead DF patients to endure sluggishly healing wounds and bleak prognoses. Consequently, expediting wound closure and bolstering recuperation to curtail amputations emerge as quintessential objectives in DF management [13].

In China, the application of TCM has been investigated for its potential in detoxification, blood stasis elimination, enhancement of blood circulation, and promotion of wound healing, whether administered orally or topically, independently or in conjunction with conventional Western medicine. Therefore, TCM has been extensively utilized in the treatment of various types of wounds, particularly DFUs [12–14]. These herbs are often used in the form of decoctions. Among them, Yanghe decoction is a traditional remedy for the treatment of necrosis in TCM, which has the effect of warming yang and dispersing cold, replenishing blood and resolving stagnation, and may potentially improve the intractable state of DF ulcers and shorten the recovery time of patients’ foot wounds [15–18]. Yanghe decoction comes from the “Surgical evidence and treatment of the whole life set” by the famous Qing Dynasty physician Wang Weide and is composed of Rehmannia root, ephedra, deer antler gum, cinnamon, cannon ginger charcoal, raw licorice, and semen brassicae. TCM physicians believe that the cause of the yin syndrome treated by Yanghe decoction is mostly due to the lack of yang qi and blood in the body, which leads to the condensation of cold and evil in the body, and for a long time, it merges with the evil of phlegm and dampness and is blocked in the blood veins, resulting in the loss of nourishment to muscles, bones, and tendons [19]. Yanghe decoction can support yang qi and warm qi and blood, which fully embodies the idea of TCM to support yang. The results of Zhou et al. [20] suggest that Yanghe decoction combined with modern medical therapy in the treatment of DF may improve the nerve conduction function of patients and enhance the therapeutic effect. Yanghe decoction is also widely used in the treatment of DF in clinical practice, and related clinical trials are also reported frequently, so this meta‐analysis systematically evaluated the potential effect of Yanghe decoction on DF, hoping to provide preliminary evidence for the exploration of TCM in DF treatment.

## 2. Method

This meta‐analysis followed Preferred Reporting Items for Systematic Reviews and Meta‐Analyses (PRISMA) guidelines [21]. The PRISMA checklist is available in the Supporting Information. We registered this meta‐analysis on the PROSPERO with registration number CRD42022369556.

### 2.1. Search Strategy

The databases included Web of Science, PubMed, The Cochrane Library, Embase, CNKI, Wanfang, and VIP databases, and randomized controlled trials (RCTs) were searched from their establishment to December 6, 2025. The key search terms were “Yanghe decoction” AND (“diabetic foot” OR “diabetic foot ulcer”) AND (“randomized controlled trial” OR “RCT” OR “randomized clinical trial” OR “randomly”). The search strategy was adjusted appropriately for different databases to ensure comprehensiveness.

### 2.2. Eligibility and Exclusion Criteria

#### 2.2.1. Types of Studies

Published and unpublished RCTs were included in this meta‐analysis. We excluded nonrandomized studies as they are associated with a high risk of bias.

#### 2.2.2. Types of Participants

All the patients included in the study met the Western medical diagnostic criteria of the Chinese Guidelines for the Prevention and Treatment of Type 2 Diabetes (2020 Edition) [22], regardless of gender, age, and duration of DM and DF, and without mental abnormalities and other serious systemic diseases.

#### 2.2.3. Interventions

Treatment group: Yanghe decoction or its addition or subtraction + conventional treatments such as surgical debridement, anti‐infection, and other Chinese medicine decoctions. Control group: conventional treatments such as surgical debridement, anti‐infection, and other Chinese medicine decoctions. Other interventions used in the treatment and control groups were approximately the same.

#### 2.2.4. Types of Outcome Measures

Studies had to include at least one of the following indicators: (1) total efficiency, (2) ankle‐brachial index (ABI), (3) wound healing rate after treatment, and (4) wound healing time.

#### 2.2.5. Exclusion Criteria

(1) Duplicate literature, (2) studies using animals or other types of studies such as self‐control and retrospective studies, (3) the results of the studies lacked corresponding inclusion indicators, and (4) there were other influencing factors that interfered with the study results.

### 2.3. Literature Screening and Data Extraction

Two reviewers independently screened the literature by reading the titles, abstracts, and full texts according to the prespecified eligibility criteria. Any discrepancies were resolved through discussion with a third senior reviewer. The following data were extracted from the included studies: title, publication date, study design, first author, basic characteristics of participants, intervention measures, risk of bias assessment, and outcome indicators. For important data not clearly reported in the literature, attempts were made to contact the original authors for supplementation.

### 2.4. Literature Quality Evaluation

Risk of bias was assessed for included studies according to the “Risk of bias” tool recommended in the Cochrane Handbook [23]. The evaluation indicators included random sequence generation, allocation concealment, blinding of participants and personnel, blinding of outcome assessment, incomplete outcome data, selective reporting, and other sources of bias. Each indicator was rated as low risk, unclear risk, or high risk. Cross‐checks were performed after the evaluation, and discrepancies were resolved by a third reviewer.

### 2.5. Statistical Methods

All statistical analyses were performed using RevMan 5.4 and Stata 15.0 software. Count data were expressed as relative risk (RR) with 95% confidence interval (CI), and continuous data were expressed as mean difference (MD) or standardized mean difference (SMD) with 95% CI, according to the homogeneity of outcome indicators. Heterogeneity among studies was assessed using the Cochrane Q test and *I* ^2^ statistic: an *I* ^2^ value > 50% indicated substantial heterogeneity, and a random‐effects model was used for meta‐analysis; otherwise, a fixed‐effects model was applied. Descriptive analysis or sensitivity analysis was used to explore the sources of significant heterogeneity. Publication bias was evaluated using funnel plots and Egger’s test; if publication bias was identified, the nonparametric trim‐and‐fill method was used to further assess its impact on the meta‐analysis results. A two‐sided *p* < 0.05 was considered statistically significant.

## 3. Result

### 3.1. The Process and Results of Screening Literature

**FIGURE 1:**
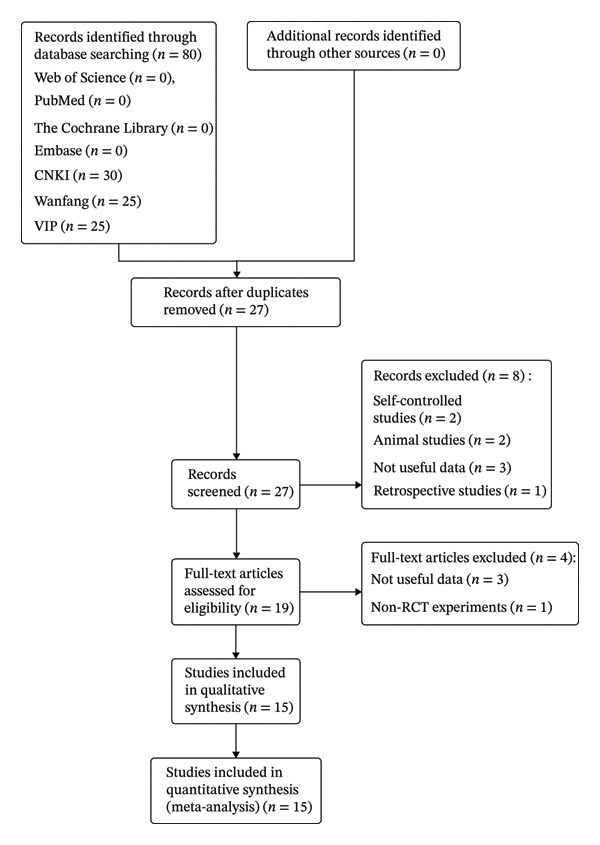
A flowchart summarizing all study assessment processes.

A preliminary search found 80 relevant articles, including 53 duplicate publications, eight articles were removed after browsing article titles and abstracts, and four articles were screened again after reading the full text. Fifteen literature studies [15, 17, 18, 24–35] with 1224 patients were included, including 614 in the treatment group and 610 in the control group. The literature screening process and results are shown in Figure 1, and papers included in the current meta‐analysis are shown in Table 1.

**TABLE 1:** Papers included in the current meta‐analysis.

Study	Number of cases		Age		Interventions		Available outcomes
	T	C	T	C	T	C	
Chen 2012	14	14	66.9 ± 10.6	64 ± 12.4	Yanghe decoction or its addition or subtraction + other treatments	Other treatments	Total efficiency, wound healing rate after treatment
Feng 2021	50	50	53.11 ± 6.38	53.69 ± 6.74	Yanghe decoction or its addition or subtraction + other treatments	Other treatments	Total efficiency, wound healing time
Guo 2016	30	30	51.27 ± 7.86	50.44 ± 6.14	Yanghe decoction or its addition or subtraction + other treatments	Other treatments	Total efficiency, ABI
Guo 2021	30	30	56.75 ± 5.46	56.28 ± 5.81	Yanghe decoction or its addition or subtraction + other treatments	Other treatments	Total efficiency
Jin 2018	30	30	54.40 ± 1.46	53.83 ± 1.76	Yanghe decoction or its addition or subtraction + other treatments	Other treatments	Total efficiency, wound healing rate after treatment
Li 2022	45	45	60.54 ± 5.53	62.37 ± 5.79	Yanghe decoction or its addition or subtraction + other treatments	Other treatments	Total efficiency, wound healing time
Liang 2016	41	37	53.61 ± 9.22	54.13 ± 8.64	Yanghe decoction or its addition or subtraction + other treatments	Other treatments	Total efficiency, wound healing rate after treatment, wound healing time
Lin 2018	40	40	59.13 ± 4.62	58.34 ± 4.29	Yanghe decoction or its addition or subtraction + other treatments	Other treatments	Total efficiency
Liu 2013	30	30	62.9	70.1	Yanghe decoction or its addition or subtraction + other treatments	Other treatments	Total efficiency, ABI
Long 2019	40	40	49.27 ± 7.96	48.54 ± 7.12	Yanghe decoction or its addition or subtraction + other treatments	Other treatments	Total efficiency, ABI
Peng 2018	44	44	55.99 ± 7.44	56.15 ± 7.13	Yanghe decoction or its addition or subtraction + other treatments	Other treatments	Total efficiency, wound healing rate after treatment
Wang 2008	40	40	68.1	67.3	Yanghe decoction or its addition or subtraction + other treatments	Other treatments	Total efficiency, wound healing time, wound healing rate after treatment
Wang 2021	55	55	58.54 ± 10.38	63.48 ± 12.32	Yanghe decoction or its addition or subtraction + other treatments	Other treatments	Total efficiency, wound healing time
Xu 2016	35	35	55.36 ± 8.72	54.38 ± 9.89	Yanghe decoction or its addition or subtraction + other treatments	Other treatments	Total efficiency
Zhang 2017	90	90	59.8 ± 4.2	58.9 ± 3.9	Yanghe decoction or its addition or subtraction + other treatments	Other treatments	Total efficiency

*Note:* T: treatment group; C: control group. Other treatments: conventional treatments such as surgical debridement, anti‐infection, and other Chinese medicine decoctions.

Abbreviation: ABI, ankle‐brachial index.

### 3.2. Inclusion in the Quality Evaluation of Literature

**FIGURE 2:**
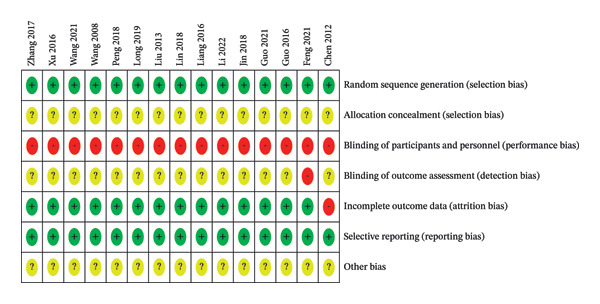
Risk of bias map for inclusion literature.

**FIGURE 3:**
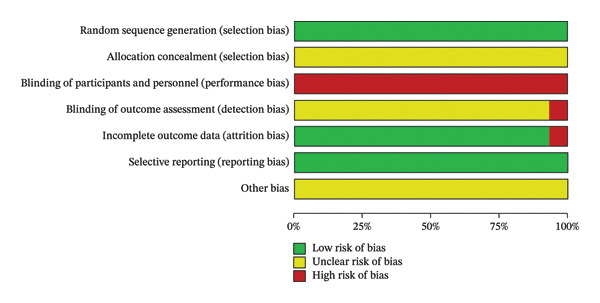
Risk of bias plot.

The quality of the included RCTs was assessed by two reviewers according to the “Risk of bias” tool recommended by the Cochrane Collaboration’s. The included studies were randomized and evenly distributed between groups. Five of these studies [15, 30–33] were grouped using a random table of numbers, four [24, 26, 28, 34] were randomized by order of presentation, one study [17] was randomized by lottery, and none of the remaining trials described a specific grouping method. None of the included studies were blinded or reported on allocation concealment, and the completeness of all data results was judged as low risk of bias as the specified indicators were adequately reported and it was not possible to judge whether the included studies were selective in reporting findings and whether there were other sources of bias. None of the included studies mentioned whether follow‐up was required. The results of the risk of bias assessment are shown in Figures 2 and 3.

### 3.3. Total Efficiency

**FIGURE 4:**
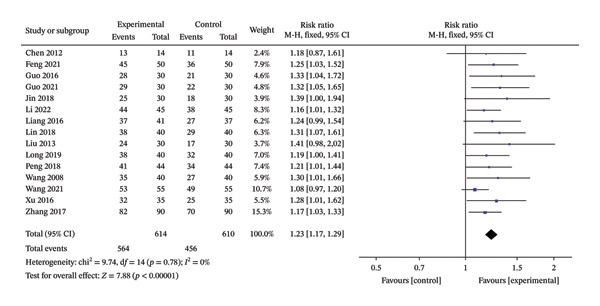
Meta‐analysis forest plot of the total efficiency.

Fifteen RCTs, including 1224 patients, were included [15, 17, 18, 24–35]. The heterogeneity results showed *p* = 0.78 and *I* ^2^ = 0%, suggesting that the heterogeneity was small and a fixed‐effect model was adopted. Results showed that the total response rate of the treatment group was higher than that of the control group, and the difference was statistically significant (RR = 1.23, 95% CI: 1.17 to 1.29, *p* < 0.00001). The results are shown in Figure 4.

### 3.4. Wound Healing Time

**FIGURE 5:**
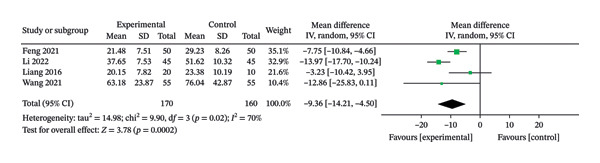
Meta‐analysis forest plot of the wound healing time.

Four RCTs [15, 24, 25, 30], involving 330 patients, were included in this review. The heterogeneity results showed *p* = 0.02 and *I* ^2^ = 70%, suggesting greater heterogeneity and using a random‐effects model. The results showed that the treatment group had a potential advantage over the control group in terms of wound healing rate after treatment, and the difference was statistically significant (MD = −9.36, 95% CI: −14.21 to −4.50, *p* = 0.0002). The results are shown in Figure 5.

### 3.5. ABI

**FIGURE 6:**
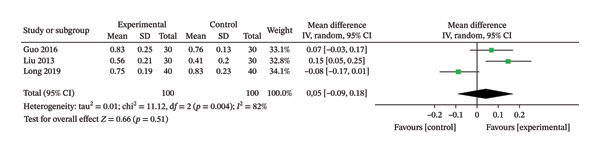
Meta‐analysis forest plot of the ABI.

Three RCTs [32, 34, 35], involving 200 patients, were included in this review. The heterogeneity results showed *p* = 0.004 and *I* ^2^ = 82%, suggesting greater heterogeneity and using a random‐effects model. The results showed that there was no significant difference in ABI between the two groups (MD = 0.05, 95% CI: −0.09 to 0.18, *p* = 0.51). The results are shown in Figure 6.

### 3.6. Wound Healing Rate After Treatment

**FIGURE 7:**
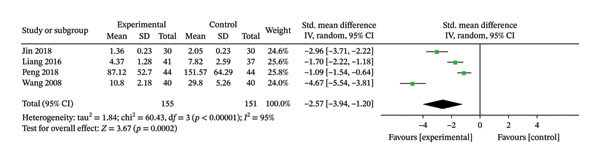
Meta‐analysis forest plot of the wound healing rate after treatment.

Four RCTs [18, 24, 26, 29], involving 306 patients, were included in this review. The heterogeneity results showed that *p* < 0.00001, *I* ^2^ = 95%, suggested that heterogeneity was large, and a random‐effects model was used. The results showed that the treatment group had a greater advantage than the control group in terms of wound healing rate after treatment, and the difference was statistically significant (SMD = −2.57, 95% CI: −3.94 to −1.20, *p* = 0.0002). The results are shown in Figure 7.

### 3.7. Publication Bias

**FIGURE 8:**
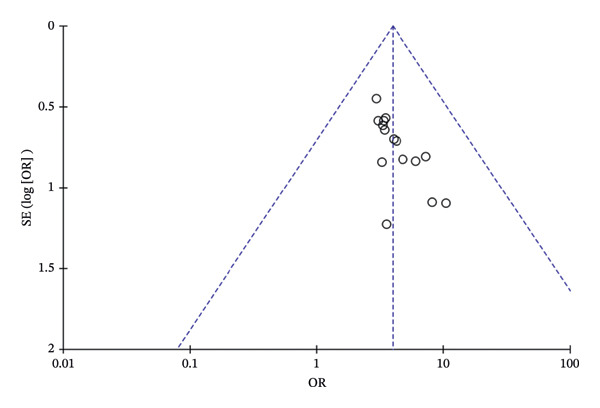
Funnel plot of publication bias test for total efficiency.

**FIGURE 9:**
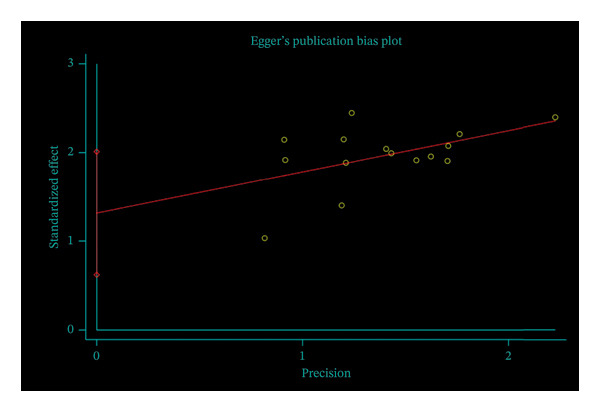
Egger’s publication bias plot.

**FIGURE 10:**
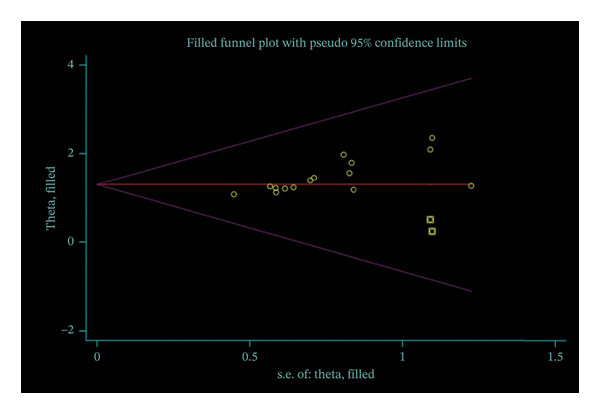
Filled funnel plot with pseudo 95% confidence limits.

Funnel plot analysis of the total effective rate showed an asymmetric distribution, suggesting the presence of publication bias (Figure 8). Egger’s test further confirmed the existence of publication bias (*p* < 0.05, 95% CI of bias: 0.62–2.01) (Figure 9). The nonparametric trim‐and‐fill method was then used to correct for publication bias, with two virtual studies added for adjustment. The pooled OR before correction was 1.348 (95% CI: 0.997–1.699, *p* < 0.01), and the corrected OR was 1.298 (95% CI: 0.956–1.640, *p* < 0.01). No significant change was observed in the 95% CI before and after correction, indicating that the meta‐analysis results were stable, and the potential publication bias did not have a substantial impact on the overall conclusions (Figure 10).

## 4. Discussion

DF is a common disabling chronic complication of diabetes, and the cause is related to the long‐term hyperglycemic state of DM patients caused by dyslipidemia, which makes lipid plaques more likely to attach and deposit on the blood vessel wall, which in turn causes lumen stenosis and affects the blood supply to the extremities, resulting in abnormal foot microcirculation [36]. In general, the pathological process of DF is very complex, involving damaged skin, muscles, tendons, bones, nerves, and blood vessels, and is accompanied by a persistent hyperglycemic state, which makes DF more difficult to heal than normal wounds, and the cost of treatment is high, and the long‐term presence of wounds will lead to the aggravation of the original disease [37]. At present, the mainstream treatment for DF mainly includes local debridement, hyperbaric oxygen, occlusive negative pressure drainage, and vascular intervention, but there are corresponding contraindications for the above treatment methods, and the course of treatment is long, and the per capita treatment cost is high [38]. Therefore, it has become a common goal of the world medical community to continuously research new ways to promote the cure of DF disease and reduce the disability and mortality rate. Studies suggest that TCM may have a potential role in the treatment of diabetes and its associated complications. Chinese herbal medicine (CHM) is a major part of TCM, and it is often used in the treatment of ailments in the form of herbal decoctions. As one of the traditional formulas, Yanghe decoction is widely used in the treatment of DF in clinical practice [19]. Some studies suggest that Yanghe decoction may improve the ischemia and hypoxia of the peripheral nerves of the limb by improving microcirculation, shorten the wound healing time of DF patients, and achieve the potential effect of promoting wound healing and improving healing [39]. Therefore, this study collected 15 RCTs to further explore the potential role of Yanghe decoction in the treatment of DF in clinical practice and critically evaluate the clinical evidence based on an evidence‐based approach to inform the current practice and guide future research on the treatment of DF with CHM.

A total of 15 RCTs were included in this study. According to the analysis results of this study, the treatment group using modified Yanghe decoction may have a higher overall effective rate in treating DF than the control group and may shorten the wound healing time to a certain extent. The difference is statistically significant (*p* < 0.05). However, after a summary analysis of the wound healing rate study results, large heterogeneity was detected (*I* ^2^ = 95%), and the meta‐analysis results showed that the ABI between the treatment group and the control group was not statistically significant (*p* > 0.05). Most importantly, the independent efficacy of Yanghe decoction for DF cannot be determined due to the high heterogeneity of cointerventions in the included studies. Therefore, the follow‐up of this study will further explore the lack of ABI improvement in the results and the possible reasons why Yanghe decoction shows potential in improving DF ulcer wounds, but the meta‐analysis results are highly heterogeneous, as well as how to standardize interventions to accurately evaluate its independent clinical effect.

### 4.1. Pharmacological Effects of Yanghe Decoction

The prescription of Yanghe decoction comes from the “Surgical Syndrome and Treatment Complete Collection,” which includes seven herbs: Rehmannia glutinosa, ephedra, antler gum, cinnamon, Paojiang charcoal, raw licorice, and white mustard seeds. The core pathological mechanisms of DF involve microcirculatory disorders, excessive local inflammatory response, nerve damage, and metabolic disorders. The pharmacological effects of the seven components of Yanghe decoction may target these pathological links, and their multitarget synergistic effects may provide a potential biological basis for promoting wound healing—while also explaining why the formula does not significantly affect ABI, which reflects macrovascular perfusion.

The key to analyzing the discrepancy between wound healing and ABI results lies in exploring the potential target of Yanghe decoction’s action: The formula may primarily regulate the microcirculation and local wound microenvironment, rather than directly targeting macrovascular organic lesions. This is supported by the specific pharmacological effects of each component.

Cinnamon: The cinnamic aldehyde contained in cinnamon can directly dilate blood vessels in the lesion area, but it mainly targets small blood vessels and capillaries involved in local microcirculation, rather than large arteries that dominate lower limb macrovascular perfusion. Meanwhile, its hydroxychalcone compounds have insulin‐like activity, regulating blood glucose and lipid metabolism [40]. This may correct the metabolic disorder that impairs microcirculation, indirectly improving local blood supply for wound healing, but does not reverse established macrovascular stenosis or atherosclerotic plaques. Notably, cinnamon also has anti‐inflammatory activity against multiple pathogenic bacteria and can synergize with antibiotics [41], which may help control local wound infection and create a favorable healing microenvironment, but this local effect does not translate to changes in systemic macrovascular perfusion.

Rehmannia glutinosa: Its blood glucose and lipid‐regulating effects may help correct metabolic disorders in diabetic patients, reducing the inhibition of wound healing by a high‐sugar environment. This may synergize with cinnamon in metabolic regulation and microcirculation improvement, laying a potential foundation for wound healing—but again, this regulation may focus on systemic metabolic balance and local microcirculation, not macrovascular function.

Antler gum: It contains animal proteins, various amino acids, peptides, and trace elements, which can increase the number of platelets, hemoglobin, and T lymphocytes, improving the body’s immunity [42]. Enhanced immunity may promote the clearance of local wound pathogens and accelerate tissue repair, but this is a systemic immunomodulatory effect that does not directly affect large blood vessel perfusion.

Other components: Paojiang’s active ingredients have antioxidant activity [43], which may reduce oxidative stress‐induced damage to microvascular endothelial cells; ephedra’s active ingredients inhibit the production of inflammatory mediators, alleviating local inflammatory responses [44]; white mustard seeds’ phenyl isothiocyanate has broad‐spectrum antibacterial activity, which may help control local wound infection; licorice inhibits capillary permeability, enhances natural killer cell activity, and exerts antibacterial and anti‐inflammatory effects [45], while also protecting damaged peripheral nerves [46]. All these effects may center on optimizing the local wound microenvironment and improving microcirculation, rather than acting on large blood vessels.

In summary, the pharmacological effects of Yanghe decoction may be concentrated on microvascular remodeling and local microenvironment optimization, which may directly address the core pathological barriers to DF wound healing (microcirculatory disorders, local inflammation, and endothelial damage). However, since these potential effects do not involve direct dilation of large blood vessels or reversal of atherosclerotic plaques (the main factors affecting ABI), the formula cannot significantly alter ABI, which reflects lower limb macrovascular perfusion. This directly answers the key question raised by the meta‐analysis: The observed improvement in wound healing may be driven by microcirculatory and local microenvironmental improvements, while the lack of ABI change is due to the formula’s lack of targeting on macrovascular lesions.

### 4.2. Interpretation of the Nonsignificant ABI Result

The results of meta‐analysis showed that there was no significant ABI between the treatment group and the control group (*p* > 0.05), and the lack of ABI improvement suggested that the effect of Yanghe decoction may be to promote the healing of DF wounds through microvascular remodeling and local microenvironment optimization, rather than directly targeting macrovascular organic lesions.

Hyperglycemia delays wound healing by increasing proinflammatory cytokine levels and decreasing growth factor levels and by reducing blood circulation and cell proliferation and migration in wounds [47]. Network pharmacological analysis showed that the main active ingredients of Yanghe decoction included quercetin, kaempferol, glycine, naringenin, β‐sitosterol, etc. As a natural flavonoid compound, quercetin has anti‐inflammatory and antioxidant properties, which can reduce the expression of inflammatory factors and exert anti‐inflammatory effects by downregulating the TNF signaling pathway [48] and can also inhibit the expression of related inflammatory factors by inhibiting the NF‐κB/PI3K‐AKT signaling pathway, thereby exerting anti‐inflammatory effects [49, 50]. Kaempferol has anti‐inflammatory, antiapoptotic, antioxidant, and antitumor effects [51] and can downregulate the PI3K/AKT signaling pathway [52], further acting on targets such as TNF and IL‐6, and regulating high‐grade glycation end‐product‐receptors (AGE‐RAGE), HIF‐1, and interleukin‐17 (IL‐17) pathways, potentially interfering with oxidative stress and metabolic disorders. Glycine has anti‐inflammatory, immunomodulatory, and cytoprotective effects [53] and can inhibit the activation of inflammatory cells by inhibiting the TLR‐NF‐κB signaling pathway, preventing the release of inflammatory mediators and hindering inflammation [54]. β‐Sitosterol inhibits the inflammatory response by inhibiting the level of oxidative stress in the blood vessels caused by high blood sugar, as well as downregulating the release of inflammatory factors.

During the healing process of DFUs, the premature and excessive apoptosis of vascular endothelial cells will reduce neoangiogenesis, and long‐term hyperglycemia will interfere with the growth factors related to vascular endothelial cells, disrupt endothelial stability, and make it difficult for ulcers to heal [55]. The results of animal experiments by Bao Yaling et al. showed that Astragalus Yanghe Tang could inhibit NF‐κB activation, reduce the serum levels of C‐reactive protein (CRP) and IL‐6 in rats with DFUs, and increase the vascular endothelial growth factor (VEGF) and hypoxia‐inducible factor‐1αby activating the PI3K/AKT signaling pathway inducible factor‐1 (HIF‐1) and wound microvascular density, which promoted transcutaneous oximetry (tcpO2) recovery and wound healing. Previous studies [56] showed that in animal models of diabetic wounds, the local application of cinnamaldehyde to the wound edge can activate the Nrf2 pathway and inhibit the oxidative stress response of the wound skin tissue.

On this basis, the study by Jin et al. [57] established a full‐thickness skin defect model on the backs of diabetic rats, showing that cinnamaldehyde can promote vascular endothelial cell migration and tube formation, inhibit apoptosis of vascular endothelial cells under high glucose conditions by downregulating the expression of proapoptotic proteins Bax and Bak, and promote the healing of ulcer wounds in diabetic rats.

The above research results indicate that the pharmacological effects of Yanghe decoction may be more focused on microvascular remodeling and local microenvironment optimization, rather than directly dilating large blood vessels or reversing atherosclerotic plaques. It may inhibit inflammation by regulating the PI3K/AKT pathway, suppress oxidative stress and apoptosis of vascular endothelial cells, improve endothelial function, and promote microvascular angiogenesis and collateral circulation formation through the HIF‐1α/VEGF pathway, meeting the local blood supply needs for wound healing. In contrast, ABI mainly reflects blood perfusion in large vessels of the lower limbs, and Yanghe decoction lacks potent components that directly target large vessel stenosis or plaques, so it can only cause a mild improvement in ABI.

Moreover, the aforementioned processes do not involve changes in ABI. Therefore, the absence of ABI improvement suggests that Yanghe decoction may potentially promote the healing of DFUs primarily by inhibiting inflammation, suppressing oxidative stress and apoptosis of vascular endothelial cells, improving endothelial function, and facilitating microvascular formation.

### 4.3. Implications of Heterogeneity

After summarizing and analyzing the results of studies on wound healing rates, considerable heterogeneity was detected (*I* ^2^ = 95%). This high heterogeneity suggests that the observed treatment effects varied across different studies, and crucially, the independent efficacy of Yanghe decoction cannot be determined due to this heterogeneity, which is likely due to differences in the specific implementation conditions of each study, especially the nonstandardized cointerventions combined with Yanghe decoction. Below are the key sources of this incompatible intervention heterogeneity, including both patient‐related factors and intervention‐related factors.

### 4.4. Heterogeneity in Baseline Characteristics of Study Subjects

Age and disease duration of patients directly affect the extent of microvascular and nerve damage, which in turn may influence the observed response to Yanghe decoction’s potential microcirculation‐improving effects—this constitutes a nonintervention‐related source of heterogeneity that interacts with the incompatible intervention regimens. For example, among the 15 included studies, Wang et al. [25] and Li et al. [15] included patients aged 30–73, Long and Liang [32] included younger patients (48–49 years old), and Chen [31] included mostly elderly patients (average age > 64 years). Age differences affect the wound healing ability and drug tolerance: Elderly patients often have more severe microcirculatory disorders and nerve damage, which may enhance the observed need for Yanghe decoction’s microcirculation‐regulating effects, while younger patients with milder damage may show less obvious therapeutic responses. In terms of disease duration, Guo [17] included patients with a minimum disease duration of 1 year, while Liu et al. [34] included patients with a disease duration of up to 40 years. Longer diabetes duration leads to more severe microvascular and nerve damage, increasing the dependence of wound healing on the formula’s potential microcirculation‐improving effects—thus amplifying the overall heterogeneity in therapeutic outcomes, especially when combined with the diverse cointerventions mentioned below.

### 4.5. Nonstandardization of Yanghe Decoction Interventions

Compared with patient baseline heterogeneity, the most critical source of incompatible intervention heterogeneity is the nonstandardized use of “Yanghe decoction or its modifications” combined with fundamentally different cointerventions in the included studies. Among the 15 studies, six [24, 26–29, 31] combined Yanghe decoction with cointerventions that have independent therapeutic effects or even opposing therapeutic principles, which directly violates the basic premise of meta‐analytic pooling—interventions in the pooled studies should be homogeneous to ensure that the pooled effect reflects the efficacy of the target intervention itself. Specifically, the cointerventions fall into four categories with distinct characteristics, each of which independently affects wound healing and confounds the accurate evaluation of Yanghe decoction’s independent efficacy.

Herbal formulas with opposing therapeutic principles: For example, some studies combined Yanghe decoction with Simiao Yong’an decoction [26, 28, 31]. Yanghe decoction is a formula with the effect of warming yang and dispelling cold, which is suitable for DF with yang deficiency and cold coagulation syndrome, while Simiao Yong’an decoction is a formula with the effect of cooling and clearing heat, which is suitable for syndromes with heat‐toxin accumulation. The opposing therapeutic principles of the two formulas mean that their combined use changes the core therapeutic direction of Yanghe decoction, making it impossible to distinguish whether any observed therapeutic effect comes from Yanghe decoction, Simiao Yong’an decoction, or their interaction.

Modern wound care technologies with independent prohealing effects: Some studies combined Yanghe decoction with recombinant human epidermal growth factor [24], negative pressure drainage technology [25], or silver ion dressings. These modern technologies have clear independent effects on promoting wound healing: Recombinant human epidermal growth factor can directly promote epithelial cell proliferation and migration; negative pressure drainage can clear wound exudate and improve local microenvironment; silver ion dressings have strong antibacterial effects. These cointerventions can independently improve wound healing rates, which means the observed effect cannot be attributed to Yanghe decoction alone.

Topical Chinese medicine preparations: Studies such as by Pang [29] and Wang [27] combined oral Yanghe decoction with external application of Qufu Shengji San or Hongyou Ointment. These topical TCM preparations have direct effects on wound repair, and their local therapeutic effects are independent of oral Yanghe decoction, further confounding the evaluation of Yanghe decoction’s independent efficacy.

TCM nursing interventions: Two studies [32, 33] combined oral Yanghe decoction with TCM foot baths and foot massages. These nursing interventions can directly improve local microcirculation of the lower limbs and relieve nerve damage, which may have synergistic effects with Yanghe decoction’s potential microcirculation‐regulating effect, but also make it difficult to isolate the independent effect of Yanghe decoction.

The high heterogeneity severely restricts the confidence in the pooled estimate (SMD = −2.57), and the estimate cannot be regarded as a reflection of Yanghe decoction’s independent clinical efficacy. The observed effects associated with Yanghe decoction in the treatment of DF are not a single “true” effect but a complex set of results influenced by multiple factors including cointerventions and patient baseline characteristics. Therefore, the treatment‐related effects summarized in this study are more appropriately interpreted as a potential benefit highly dependent on specific scenarios. Whether any potential therapeutic effect is manifested and the intensity of the effect may be closely related to the undefined specific conditions mentioned above, rather than a stable and repeatable universal treatment effect of Yanghe decoction alone. Thus, when interpreting the results of this study and applying them to clinical practice or subsequent research, the uncertainty brought by this heterogeneity must be fully considered. Clinical decisions should not be made blindly based on the pooled effect size. Further subgroup analysis, sensitivity analysis, or targeted research that standardizes interventions and controls cointervention variables should be conducted to identify the key variables affecting the observed treatment effects and more precisely explore the applicable scope and potential value of Yanghe decoction.

Due to the heterogeneity of the research results, the credibility of the conclusion is also somewhat limited. In addition, because the potential benefits of Yanghe decoction are based on precise matching, individualized combinations of specific intervention plans are required in clinical application. For example, for early mild cases, oral Yanghe decoction combined with baths might be explored, while for moderate to severe cases, negative pressure technology, antibacterial dressings, and recombinant human epidermal growth factor might be combined to enhance local efficacy—but such combinations make it impossible to assess the independent effect of Yanghe decoction itself.

However, due to the small number of studies that can be included so far, and the fact that most of the studies are not partially blinded, there may be potential issues of selectivity and measurement bias that may affect the authenticity of the results, and all conclusions from this study need to be treated with extreme caution. Although this meta‐analysis explores the potential of Yanghe decoction as a candidate for DF treatment in combination with other therapies, there are some limitations: (1) There were certain differences in the treatment duration and outcome indicators of the various studies included in the literature, which were prone to selection bias; (2) all included studies were Chinese literature and may have linguistic bias; (3) due to the small number of literature included in the relevant studies on the treatment of DF and the possibility of selective publication of the included literature, this study may have some publication bias; and (4) all of the trials were mentioned for grouping using random methods; however, no studies described specific blinded methods for participants and researchers. Therefore, potential performance bias and detection biases were caused by insufficient and lack of blinding. In summary, more high‐quality RCTs may still be needed to further validate the conclusions of this study. At present, many treatment regimens are mostly Yanghe decoction combined with other treatment regimens for the treatment of DF, and in the future, a single application of Yanghe decoction can be used to intervene in DF, so as to provide more scientific data reference. This study systematically summarized, analyzed, and evaluated the published RCTs related to the treatment of DF by Yanghe decoction and found that the external application of Yanghe decoction has a good overall efficacy in the treatment of DF and discussed some limitations of the existing meta‐analysis, such as all trials were from China and most of the studies were mentioned to use a random method for grouping, but no studies described specific blinding methods for participants and investigators, and were designed for follow‐up clinical studies. At the same time, this study discussed the clinical application of the external treatment method of TCM represented by Yanghe decoction in the prevention and treatment of DF through the combination of TCM theory and modern medical research and gave full play to the unique advantages of TCM in the prevention and treatment of DF, which is conducive to reducing the amputation rate of diabetic patients, improving the quality of life of patients, alleviating the family and socioeconomic burden, and providing new ideas and methods for follow‐up clinical research design and TCM prevention and treatment of DF.

Future research mainly focuses on the following two aspects: On the one hand, it is found from the literature retrieved that there are relatively few basic studies on the pharmacodynamic substances and animal experiments of Yanghe decoction [19]. Therefore, future research should further explore the material basis and pharmacodynamic material basis of the curative effect of the prescription on the basis of the nature of the syndrome, establish the correlation between the pharmacological effect and clinical application, and deeply study the mechanism of action of Yanghe decoction in the treatment of diseases, so as to provide a more reliable basis for the efficacy of Yanghe decoction in the intervention of DF. On the other hand, it is necessary to give full play to the advantages of TCM, combine TCM with the emerging DF therapy, and further carry out clinical research on the combination of TCM preparations and new therapies for the treatment of DF represented by Yanghe decoction. For example, some advanced assistive technologies, such as hyperbaric oxygen therapy, local pressurized oxygen therapy, negative pressure wound treatment, and tibial transverse transport, can accelerate the healing of DF wounds and effectively shorten the length of hospital stay. In recent years, nanotechnology has also become one of the research hotspots in the treatment of diabetic patients and their related complications, and studies have confirmed that nanomaterials play a significant role in promoting wound vascular proliferation, nerve repair, inhibiting inflammatory response and anti‐infection [58]. However, the current research on the combination of local drugs and systemic administration of nanomaterials in the treatment of DF is still relatively weak, and clinical research on the combination of Yanghe decoction and nanomaterials in the treatment of DF can be carried out in the future, so as to provide new targets for the treatment of DF with the combination of traditional Chinese and Western medicine. In addition, considering that patients with DF often have comorbidities such as anxiety, depression, and sleep disorders, which may negatively affect wound healing and treatment compliance, future research could also explore the potential role of Yanghe decoction in neural regulation. A recent comprehensive study on plant‐derived drugs has shown that natural products with neuropharmacological activity can exhibit antianxiety, sedative, and antidepressive effects in animal models, and their mechanisms of action may be related to the regulation of targets such as the serotonin transporter [59]. Therefore, further investigation into whether Yanghe decoction has similar neural regulatory functions and whether this effect can help improve the overall prognosis of patients with DF is expected to provide a new research perspective for the clinical application of Yanghe decoction.

## Author Contributions

Xia Wang and Qiu Chen conceived the study, designed the search strategy, and conducted the study selection. Xia Wang and Yunjiao Yang extracted the data, performed the statistical analyses, and evaluated the risk of bias of the included studies. Xia Wang drafted the manuscript and processed the pictures and tables. Qiu Chen provided the guidance and resolved disagreements.

## Disclosure

All authors read and approved the final version of the manuscript.

## Ethics Statement

The authors have nothing to report.

## Consent

The authors have nothing to report.

## Conflicts of Interest

The authors declare no conflicts of interest.

## Supporting Information

PRISMA 2020 checklist.

## Supporting information


**Supporting Information** Additional supporting information can be found online in the Supporting Information section.

## Data Availability

The original contributions presented in this study are included in the article or Supporting Information; further inquiries can be directed to the corresponding author.
